# Closed-Loop Recycling Dual-Mode Counter-Current Chromatography with Specified Sample Loading Durations: Modeling of Preparative and Industrial-Scale Separations

**DOI:** 10.3390/molecules26216561

**Published:** 2021-10-29

**Authors:** Artak E. Kostanyan, Andrey A. Voshkin

**Affiliations:** Kurnakov Institute of General and Inorganic Chemistry, Russian Academy of Sciences, Leninsky Prospekt 31, 119991 Moscow, Russia; kost@igic.ras.ru

**Keywords:** closed-loop recycling chromatography, dual-mode counter-current chromatography, model-based design, separation of complex mixtures with widely different partition coefficients

## Abstract

We previously reported on a new counter-current chromatography (CCC) operating mode called closed-loop recycling dual-mode counter-current chromatography (CLR DM CCC), which incorporates the advantages of closed-loop recycling (CLR) and dual-mode (DM) counter-current chromatography and includes sequential separation of compounds in the closed-loop recycling mode with the mobile *x*-phase and in the inverted-phase counter-current mode with the mobile *y*-phase. The theoretical analysis of several implementations of this separation method was carried out under impulse sample injection conditions. This study is dedicated to the further development of CLR DM CCC theory applied to preparative and industrial separations, where high-throughput operation is required. Large sample volumes can be loaded via continuous loading within a specified time. To simulate CLR DM CCC separations with specified sample loading durations, equations are developed and presented in “Mathcad” software.

## 1. Introduction

A number of preparative methods for high-performance liquid–liquid partition chromatography, commonly known as counter-current chromatography (CCC), are widely used from laboratory to industrial scales due to the high adaptability of these methods to different separation tasks [1,2,3,4,5,6,7,8,9,10,11,12,13,14,15,16,17,18,19,20,21,22,23,24,25,26,27,28,29,30,31,32,33,34]. Recently in [6,7], a new two-stage CCC separation method called closed-loop recycling dual-mode counter-current chromatography (CLR DM CCC) was developed, which is a combination of two well-known methods—closed-loop recycling (CLR) and dual-mode (DM) counter-current chromatography. In the first stage, in the closed-loop recycling mode, the mixture compounds with low partition coefficients are separated and eluted with the mobile *x*-phase through one end of a column. In the second stage, the compounds with higher partition coefficients are separated in the inverted-phase counter-current mode and eluted with the mobile *y*-phase through the opposite end of the column.

As noted in [7], for practical implementation of closed-loop recycling dual-mode counter-current chromatography separation processes, preliminary mathematical modeling is needed. To simulate and design the CLR DM CCC separations, it is necessary to develop the mathematical description (the theory) of these processes. In [6,7], the theory of CLR DM CCC separations was developed based on the ideal [6] and non-ideal [7] recycling models for the conditions of impulse sample injection. In preparative and production separations, large volumes of the mixture to be separated must be loaded in order to obtain sufficient quantities of the corresponding products. Large volumes of feed mixtures can be loaded by increasing the sample loading time. This work is devoted to the further development of the CLR DM CCC theory in relation to the conditions for preparative and industrial-scale separations. Basing on the mathematical models presented in [6,7], new equations are derived permitting the calculation of the optimum operating parameters of the CLR DM CCC separation processes with long-term sample loading. The results of the simulation of the separations with long sample loading times demonstrate that proper selection of the sample solution loading time (operating parameter *t_s_*) can increase the productivity by an order of magnitude, providing acceptable compound separation results.

## 2. Preparative and Industrial-Scale Closed-Loop Recycling Dual-Mode Counter-Current Chromatography Separations

**Figure 1:**
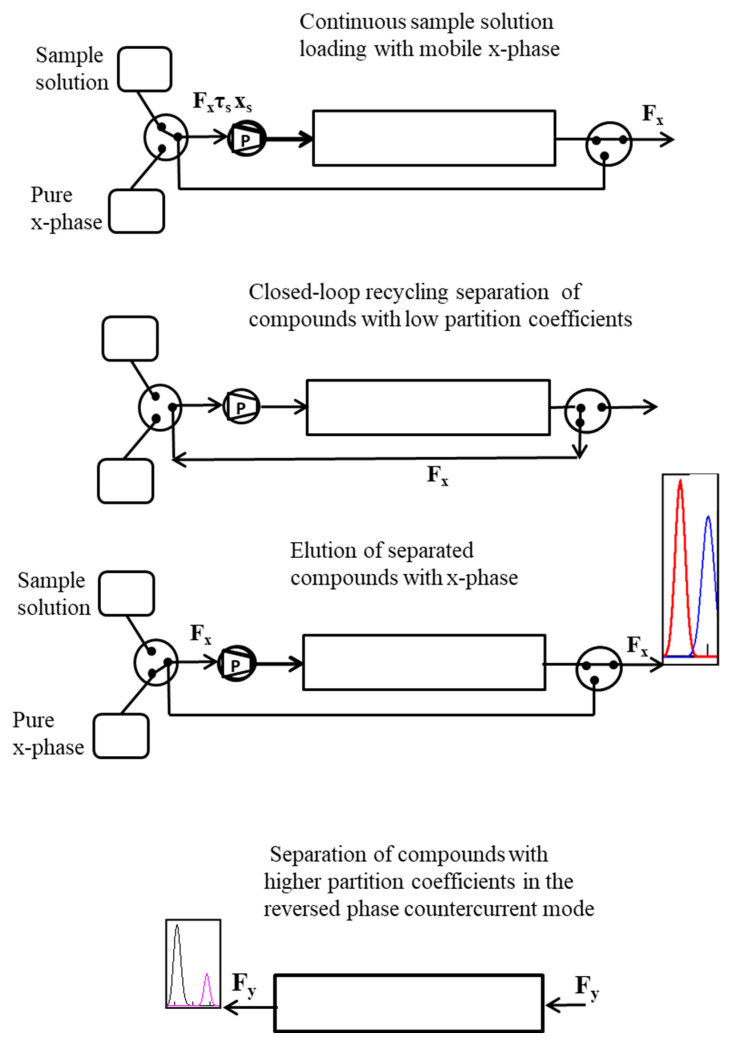
Schematic diagram of CLR DM CCC separations with specified sample loading durations: *F_x_* and *F_y_* are the volumetric flow rate of the *x*- and *y*-phase; *x_s_* is the sample concentration of the compound *K_D_*; *τ_s_* is the sample solution loading time.

As mentioned above, large sample volumes can be loaded by increasing the sample loading time, which can be achieved by replacing a sample injection with sample loops with the continuous loading of the sample within a specified time [29,30]. A schematic diagram of CLR DM CCC separations with specified sample loading durations is shown in Figure 1.

The closed-loop recycling dual-mode counter-current chromatography installation has two *x*-phase tanks: one with a pure *x*-phase and the second containing the solution of the compounds to be separated in the *x*-phase. The first stage of the CLR DM CCC is carried out in three steps: (1) continuous loading of the solution of the compounds in the *x*-phase into the system within a specified time at the same rate as the pure *x*-phase; (2) separation of compounds with low partition coefficients in closed-loop recycling mode; (3) elution of the separated compounds with the *x*-phase. At the second stage, the phases are inverted and the compounds with higher partition coefficients are separated in the counter-current mode with the mobile *y*-phase.

In this study, we will take into account the influence of the sample loading time and consider two options for a CLR DM CCC installation: one option involving short connective tubing (with a small volume of the recycling system) and one option involving long connective tubing.

## 3. Theory of Closed-Loop Recycling Dual-Mode Counter-Current Chromatography Separations with Specified Sample Loading Durations

Here, we considered the linear extraction–chromatographic separation processes and assumed that the volumes of the *x*- and *y*-phases in the column and the column efficiency, which were measured via the number of theoretical plates, were constant in both operating modes. The column efficiency does not change when the elution mode is switched, provided that the stationar*y*-phase retention is the same in both modes. This only occurs when the stationar*y*-phase retention (the fractional volume of the *y*-phase in the column) is 0.5. The results presented in this paper are valid for practical applications where the stationar*y*-phase retention is 0.5 and the sample is loaded over time (not exceeding 20% of the mean residence time).

### 3.1. First Stage of Closed-Loop Recycling Dual-Mode Counter-Current Chromatography with Specified Sample Loading Durations

The mathematical description of the first separation stage for the process scheme with the short recycling line is based on the ideal scenario, while for the process scheme with the long recycling line, this is based on the non-ideal recycling models.

#### 3.1.1. Equations Used to Simulate the First Stage of the Closed-Loop Recycling Dual-Mode Counter-Current Chromatography Separations with Specified Sample Loading Duration Based on the Ideal Recycling Model

Using the approaches we developed in our previous studies [6,7,29,30], the following equations were derived to simulate the first stage of the preparative and industrial-scale CLR DM CCC separations for the process scheme with the short recycling line:

(1) $$X\left(n,k,t\right)=\frac{\sqrt{6}aNe^{-\frac{3{\left[2N\left(n-1\right)+2k+Nat_{s}-2aNt\right]}^{2}}{2\left\{12\left[N\left(n-1\right)+k\right]+{\left(Nat_{s}\right)}^{2}\right\}}}}{\sqrt{\pi \left\{12\left[N\left(n-1\right)+k\right]+{\left(Nat_{s}\right)}^{2}\right\}}}\;$$

(2) $$X\left(n,N,t\right)=\frac{a\sqrt{6N}e^{-\frac{3N{\left[2n+at_{s}-2at\right]}^{2}}{2\left\{12n+N{\left(at_{s}\right)}^{2}\right\}}}}{\sqrt{\pi \left\{12n+N{\left(at_{s}\right)}^{2}\right\}}}\;$$

(3) $$X_{n}\left(k,t\right)=\overset{n}{\underset{i=1}{\sum }}\frac{\sqrt{6}aNe^{-\frac{3{\left[2N\left(i-1\right)+2k+Nat_{s}-2aNt\right]}^{2}}{2\left\{12\left[N\left(i-1\right)+k\right]+{\left(Nat_{s}\right)}^{2}\right\}}}}{\sqrt{\pi \left\{12\left[N\left(i-1\right)+k\right]+{\left(Nat_{s}\right)}^{2}\right\}}}\;$$

(4) $$X_{n}\left(N,t\right)=\overset{n}{\underset{i=1}{\sum }}\frac{a\sqrt{6N}e^{-\frac{3N{\left[2i+at_{s}-2at\right]}^{2}}{2\left\{12i+N{\left(at_{s}\right)}^{2}\right\}}}}{\sqrt{\pi \left\{12i+N{\left(at_{s}\right)}^{2}\right\}}}\;$$

with

(5) $$a=\frac{1}{1-S_{f}+S_{f}K_{D}}\;$$

where *X*(*n*,*k*,*t*) and *X*(*n*,*N*,*t*) describe the variations in the normalized concentration of the compound with an equilibrium distribution ratio of *K_D_* = *y/x* in the column and at the outlet of the column during the recycling process in any individual cycle *n*, disregarding the interactions of the concentration profiles from the previous cycles. The interactions of neighboring concentration profiles are accounted for in *X_n_*(*k,t*) and *X_n_(N,t)*. Equations (3) and (4) describe the variations in the compound *K_D_*-normalized concentration in the k-cell and at the outlet of the column (*k* = *N*) during the recycling process. Equation (3) describes the distribution of the compound *K_D_*-normalized concentration along the column at time *t*.

In Equations (1)–(5), the following designations are adopted: $X=x/\overset{¯}{x}\;$is the normalized concentration in the mobile *x*-phase; *x* is the actual concentration; $\bar{x}=\frac{Q}{V_{c}}=x_{s}F_{x}\tau_{s}/V_{c}$ is the mean concentration in the column; *x_s_* is the sample concentration of compound *K_D_*; *Q* = *x_s_F_x_τ_s_* is the amount of compound *K_D_* loaded during the specified sample solution loading time *τ_s_*; *F_x_* is the volumetric flow rate of the *x*-phase; *V_c_* is the column volume; *t* = *τF_x_*/*V_c_* is the dimensionless (normalized) time for the *x*-phase flow (flow start time *τ* = 0, *t* = 0); *t_s_* = *τ_s_F_x_/V_c_* is the dimensionless sample solution loading time; τ is the actual time; *N* is the number of equilibrium cells (theoretical plates) in the column; *k* is the current cell number; *S_f_* is the fractional volume of the *y*-phase in the column.

For two consecutive cycles, Equations (3) and (4) reduce to:

(6) $$X_{n}\left(k,t\right)=\;X\left(n-1,k,t\right)+X\left(n,k,t\right)\;\;$$

with

(7) $$X\left(n-1,k,t\right)=\frac{\sqrt{6}aNe^{-\frac{3{\left[2N\left(n-2\right)+2k+Nat_{s}-2aNt\right]}^{2}}{2\left\{12\left[N\left(n-2\right)+k\right]+{\left(Nat_{s}\right)}^{2}\right\}}}}{\sqrt{\pi \left\{12\left[N\left(n-2\right)+k\right]+{\left(Nat_{s}\right)}^{2}\right\}}}\;$$

and

(8) $$X_{n}\left(N,t\right)=\;X\left(n-1,N,t\right)+X\left(n,N,t\right)\;$$

with

(9) $$X\left(n-1,N,t\right)=\frac{a\sqrt{6N}e^{-\frac{3N{\left[2\left(n-1\right)+at_{s}-2at\right]}^{2}}{2\left\{12\left(n-1\right)+N{\left(at_{s}\right)}^{2}\right\}}}}{\sqrt{\pi \left\{12\left(n-1\right)+N{\left(at_{s}\right)}^{2}\right\}}}\;$$

**Figure 2:**
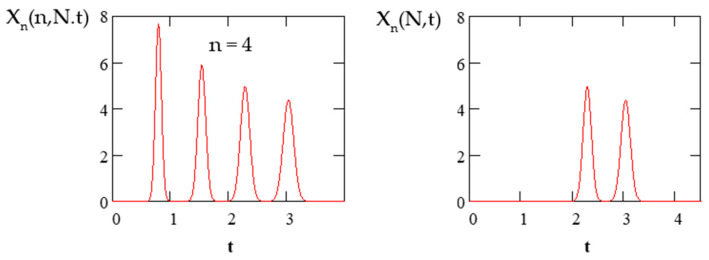
Elution profiles of compound *K_D_* = 0.5 calculated using Equations (4) and (8). *N* = 300, *S_f_* = 0.5, *t_s_* = 0.1.

These simple equations can be used to account for the overlapping of the concentration profiles of adjacent cycles during the recycling process. The difference between Equations (4) and (8) is illustrated in Figure 2. Equation (4) describes the elution profiles over the entire circulation time of the sample from the first cycle to the final cycle *n*, while Equation (8) describes the elution profiles of the last two cycles.

**Figure 3:**
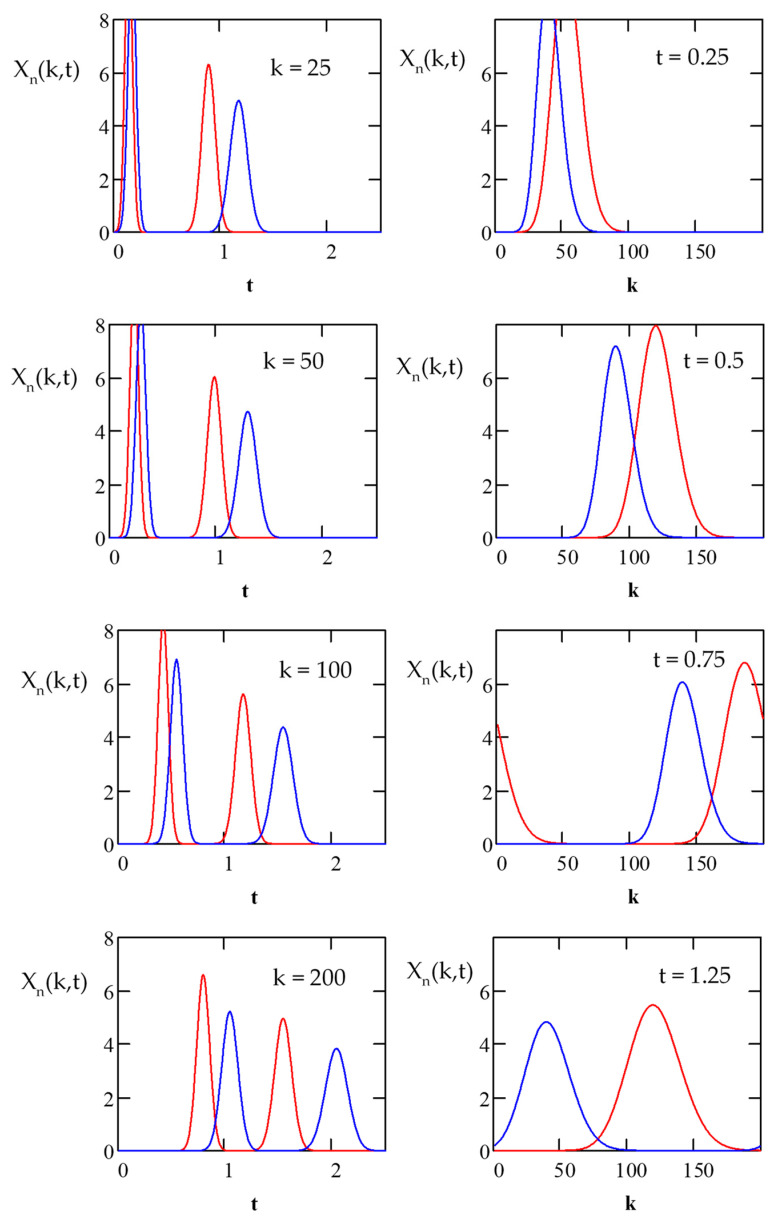
Simulation of the transport and separation of compounds *K_D_*_1_ = 0.5 and *K_D_*_2_ = 1 in the column with *N* = 200 during two cycles, calculated using Equations (1), (6), and (7). *N* = 200, *S_f_* = 0.5, *t_s_* = 0.1.

Using the above equations, the separation of compounds during the recycling process in a preparative and industrial-scale CLR CCC installation with a short recycling line can be simulated (Figure 3). Figure 3 shows an example of the simulation of the transport and separation of compounds *K_D_*_1_ = 0.5 and *K_D_*_2_ = 1 in the column with *N* = 200 during two cycles, calculated using Equations (1), (6), and (7).

Putting *t_s_* = 0 in Equations (1)–(4), (7) and (9), we obtain simpler equations for the impulse sample injection conditions:

(10) $$X\left(n,k,t\right)=\frac{aNe^{-\frac{{\left[N\left(n-1\right)+k-aNt\right]}^{2}}{2\left[N\left(n-1\right)+k\right]}}}{\sqrt{2\pi \left[N\left(n-1\right)+k\right]}}\;\;$$

(11) $$X\left(n,N,t\right)=\frac{a\sqrt{N}e^{-\frac{N{\left[n-at\right]}^{2}}{2n}}}{\sqrt{2\pi n}}\;\;$$

(12) $$X_{n}\left(k,t\right)=\overset{n}{\underset{i=1}{\sum }}\frac{aNe^{-\frac{{\left[N\left(i-1\right)+k-aNt\right]}^{2}}{2\left[N\left(i-1\right)+k\right]}}}{\sqrt{2\pi \left[N\left(i-1\right)+k\right]}}\;\;$$

(13) $$X_{n}\left(N,t\right)=\overset{n}{\underset{i=1}{\sum }}\frac{a\sqrt{N}e^{-\frac{N{\left[i-at\right]}^{2}}{2n}}}{\sqrt{2\pi i}}\;\;$$

(14) $$X\left(n-1,k,t\right)=\frac{aNe^{-\frac{{\left[N\left(n-2\right)+k-aNt\right]}^{2}}{2\left[N\left(n-2\right)+k\right]}}}{\sqrt{2\pi \left[N\left(n-2\right)+k\right]}}\;\;$$

(15) $$X\left(n-1,N,t\right)=\frac{aNe^{-\frac{{\left[N\left(n-1\right)-aNt\right]}^{2}}{2N\left(n-1\right)}}}{\sqrt{2\pi N\left(n-1\right)}}\;\;$$

Equations (6) and (8) remain valid.

#### 3.1.2. Equations Used to Simulate the First Separation Stage of the Closed-Loop Recycling Dual-Mode Counter-Current Chromatography with Specified Sample Loading Durations Based on the Non-Ideal Recycling Model

Similar to Equations (1)–(4), (7) and (9) based on the non-ideal recycling model, the following equations were derived to simulate the first separation stage of CLR DM CCC with specified sample loading durations:

(16) $$X\left(n,k,t\right)=\frac{aN\sqrt{6N_{ec}}e^{-\frac{3N_{ec}}{2}\frac{{\left[2N\left(n-1\right)+2k+2aNb\left(n-1\right)+aNt_{s}-2aNt\right]}^{2}}{\left[12\left[N\left(n-1\right)+k\right]+{\left(Nat_{s}\right)}^{2}\right]N_{ec}+12N^{2}a^{2}\left(n-1\right)b^{2}}}}{\sqrt{\pi }\sqrt{\left[12\left[N\left(n-1\right)+k\right]+{\left(Nat_{s}\right)}^{2}\right]N_{ec}+12N^{2}a^{2}\left(n-1\right)b^{2}}}\;$$

(17) $$X\left(n,N,t\right)=\frac{a\sqrt{6NN_{ec}}e^{-\frac{3N_{ec}N{\left[2n+a\left(2b\left(n-1\right)+t_{s}-2t\right)\right]}^{2}}{2\left\{\left[12n+N{\left(at_{s}\right)}^{2}\right]N_{ec}+12Na^{2}\left(n-1\right)b^{2}\right\}}}}{\sqrt{\pi }\sqrt{\left[12n+N{\left(at_{s}\right)}^{2}\right]N_{ec}+12Na^{2}\left(n-1\right)b^{2}}}\;$$

(18) $$X_{n}\left(k,t\right)=\overset{n}{\underset{i=1}{\sum }}\frac{aN\sqrt{6N_{ec}}e^{-\frac{3N_{ec}}{2}\frac{{\left[2N\left(i-1\right)+2k+aN\left(2b\left(i-1\right)+t_{s}-2t\right)\right]}^{2}}{\left[12\left[N\left(i-1\right)+k\right]+{\left(Nat_{s}\right)}^{2}\right]N_{ec}+12N^{2}a^{2}\left(i-1\right)b^{2}}}}{\sqrt{\pi }\sqrt{\left[12\left[N\left(i-1\right)+k\right]+{\left(Nat_{s}\right)}^{2}\right]N_{ec}+12N^{2}a^{2}\left(i-1\right)b^{2}}}\;\;$$

(19) $$X_{n}\left(N,t\right)=\overset{n}{\underset{i=1}{\sum }}\frac{a\sqrt{6NN_{ec}}e^{-\frac{3N_{ec}N{\left[2i+a\left(2b\left(i-1\right)+t_{s}-2t\right)\right]}^{2}}{2\left\{\left[12i+N{\left(at_{s}\right)}^{2}\right]N_{ec}+12Na^{2}\left(i-1\right)b^{2}\right\}}}}{\sqrt{\pi }\sqrt{\left[12i+N{\left(at_{s}\right)}^{2}\right]N_{ec}+12Na^{2}\left(i-1\right)b^{2}}}\;\;$$

(20) $$X\left(n-1,k,t\right)=\frac{aN\sqrt{6N_{ec}}e^{-\frac{3N_{ec}}{2}\frac{{\left[2N\left(n-2\right)+2k+2aNb\left(n-2\right)+aNt_{s}-2aNt\right]}^{2}}{\left[12\left[N\left(n-2\right)+k\right]+{\left(Nat_{s}\right)}^{2}\right]N_{ec}+12N^{2}a^{2}\left(n-2\right)b^{2}}}}{\sqrt{\pi }\sqrt{\left[12\left[N\left(n-2\right)+k\right]+{\left(Nat_{s}\right)}^{2}\right]N_{ec}+12N^{2}a^{2}\left(n-2\right)b^{2}}}\;\;$$

(21) $$X\left(n-1,N,t\right)=\frac{aN\sqrt{6N_{ec}}e^{-\frac{3N_{ec}}{2}\frac{{\left[2N\left(n-1\right)+2aNb\left(n-2\right)+aNt_{s}-2aNt\right]}^{2}}{\left[12N\left(n-1\right)+{\left(Nat_{s}\right)}^{2}\right]N_{ec}+12N^{2}a^{2}\left(n-2\right)b^{2}}}}{\sqrt{\pi }\sqrt{\left[12N\left(n-1\right)+{\left(Nat_{s}\right)}^{2}\right]N_{ec}+12N^{2}a^{2}\left(n-2\right)b^{2}}}\;\;$$

where *N_ec_* is the number of perfectly mixed cells quantifying the dispersion in the recycling system, and *b = V_ec_/V_c_* is the ratio of the column volume *V_c_* to the volume of the recycling system *V_ec_*.

Equations (6) and (8) remain valid.

### 3.2. Equations to Simulate the Second Stage of Closed-Loop Recycling Dual-Mode Counter-Current Chromatography Separations with Specified Sample Loading Durations

To describe the variations in compound concentrations at the opposite outlet of the column (*k =* 1) during the second stage of CLR DM CCC separations, the following equation can be used [6,7]:

(22) $$\begin{array}{cc} Y\left(t,1\right)= & K_{D}e^{-K_{D}aNt}\overset{50}{\underset{i=1}{\sum }}\frac{{\left(K_{D}aNt\right)}^{k-1}}{\left(k-1\right)!}X_{n}\left(k,t_{x}\right)\\ & +K_{D}\;\overset{N}{\underset{i=51}{\sum }}\frac{{\left(K_{D}aNt\right)}^{k-1}e^{k-1-K_{D}aNt}}{\left(k-1\right)\sqrt{2\pi \left(k-1\right)}}X_{n}\left(k,t_{x}\right) \end{array}$$

where $Y=y/\bar{x}$ is the dimensionless concentration of a compound in the *y*-phase and $t=\tau F_{y}/V_{c}$ is the dimensionless time for the *y*-phase flow (flow start time *τ* = 0, *t* = 0); *F_y_* is the volumetric flow rate of the *y*-phase; *X_n_*(*k*,*t_x_*) describes the distribution of the compound *K_D_* along the column at the end of the first stage of separation (*τ* = *τ_x_*, *t* = *t_x_*), which is determined by Equations (3) and (18) with *t* = *t_x_* ($t_{x}=\tau_{x}F_{x}/V_{c}$).

Using the equations presented above in Section 3.1, various options for CLR DM CCC separation with specified sample loading durations can be designed and simulated. Let us consider separations of mixtures containing compounds with lower and higher partition coefficients.

## 4. Separation of Complex Mixtures Containing Compounds with Lower and Higher Partition Coefficients Using Closed-Loop Recycling Dual-Mode Counter-Current Chromatography with Specified Sample Loading Durations

**Figure 4:**
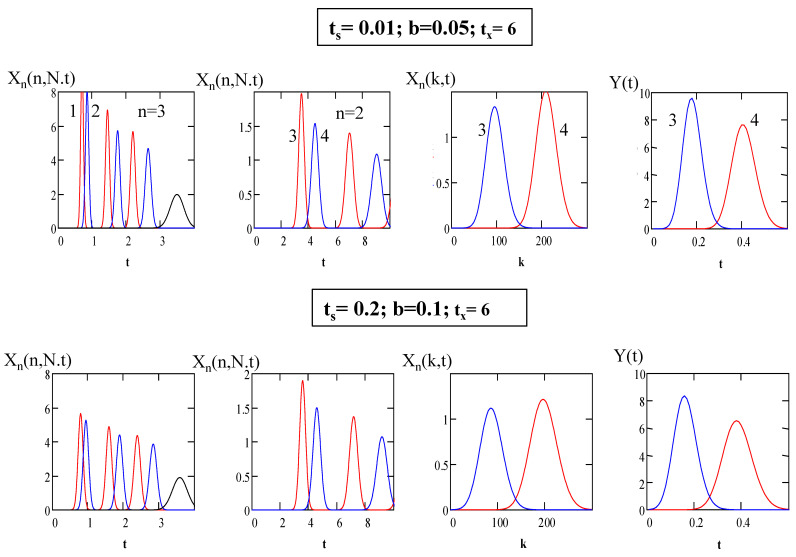
Simulation of the separation of the mixture containing four compounds (*K_D_*_1_ = 0.4, *K_D_*_2_ = 0.7, *K_D_*_3_ = 6, *K_D_*_4_ = 8) on the closed-loop setup with *N* = 300, *N_ec_* = 200, and *S_f_* = 0.5.

**Figure 5:**
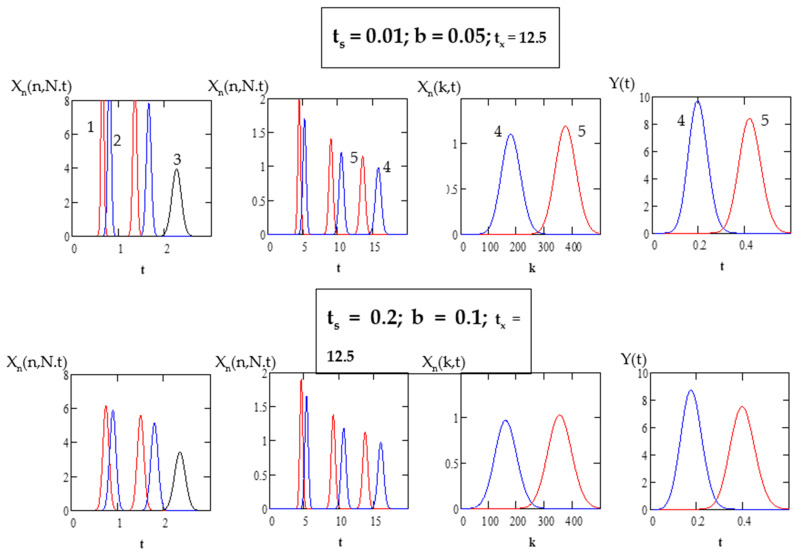
Simulation of the separation of the mixture containing five compounds (*K_D_*_1_ = 0.3, *K_D_*_2_ = 0.6, *K_D_*_3_ = 3.5, *K_D_*_4_ = 8, *K_D_*_5_ = 9.5) on the closed-loop setup with *N* = 500, *N_ec_* = 200, and *S_f_* = 0.5.

To simulate CLR DM CCC separation processes based on the above analytical expressions, it is convenient to use standard computer programs. Figure 4 and Figure 5 show several simulation examples of the separation of complex mixtures containing compounds with lower and higher partition coefficients using the “Mathcad” program.

Figure 4 shows two versions of the separation process for the mixture containing four compounds (*K_D_*_1_ = 0.4, *K_D_*_2_ = 0.7, *K_D_*_3_ = 6, *K_D_*_4_ = 8) on the closed-loop setup with *N* = 300, *N_ec_* = 200, *S_f_* = 0.5 and short (*b* = 0.05) and long (*b* = 0.1) recycling lines under two different sample loading conditions: *t_s_* = 0.01 and *t_s_* = 0.2. In the first stage, after three passages through the column, the compounds with low partition coefficients (1 and 2) are separated via the mobile *x*-phase, while in the second stage, the compounds with higher partition coefficients (3 and 4) are separated in the inverted-phase counter-current mode with the mobile *y*-phase. The separation of the compounds in closed-loop recycling is simulated by Equation (19). After the third cycle of recycling of compounds 1 and 2 at *t* = 2 and *t* = 2.2, the loop is opened and the separated fractions of compounds 1 and 2 are eluted with the *x*-phase. After the elution of these compounds at *t* = 3 and *t* = 3.1, the loop is closed again and compounds 3 and 4 continue to be recycled until *t* = *t_x_* = 6. The concentrations of compounds 3 and 4 in the column are simulated using Equation (18), putting *t* = *t_x_* = 6. Then, the loop is opened and the second separation stage for compounds 3 and 4 in the inverted-phase counter-current mode with the mobile *y*-phase starts. The elution profiles of these compounds are simulated by Equation (22).

Figure 5 shows two versions of the separation process for the mixture containing five compounds (*K_D_*_1_ *=* 0.3, *K_D_*_2_ = 0.6, *K_D_*_3_ *=* 3.5, *K_D_*_4_ = 8, *K_D_*_5_ = 9.5) on the closed-loop setup with *N* = 500, *N_ec_* = 200, *S_f_* *=* 0.5 and short (*b =* 0.05) and long (*b =* 0.1) recycling lines under two sample loading conditions: *t_s_* = 0.01 and *t_s_* = 0.2. In the first stage, after two passages through the column for compounds 1 and 2 and one passage for compound 3, these compounds are separated and eluted with the *x*-phase. After the elution of these compounds the loop is closed again, and compounds 4 and 5 continue to be recycled three times (*n* = 3) until *t* = *t_x_* = 12.5. Then, the loop is opened and compounds 4 and 5 are eluted in the inverted-phase counter-current mode with the *y*-phase.

As follows from the Figure 4 and Figure 5, in the CLR DM CCC processes with specified sample loading duration, fractions of the separated solutes with high partition coefficients are about 5–10 times more concentrated than in the conventional CLR CCC processes with specified sample loading duration, and the solvent consumption is greatly reduced. The method with specified sample loading duration provides much higher process productivity than the impulse injection method. In addition, this method makes it possible to obtain concentrated fractions of the separated compounds.

Note that in the first separation stage, the separation quality is improved due to the repeated use of the column; however, after a certain number of cycles, the chromatograms of neighboring cycles begin to overlap. Due to the time delay in the CLR CCC setups with the long recycling system (with a large parameter b), the resolution between chromatograms of adjacent cycles is greater, which allows an increase in the number of passages of the sample through the column without overlapping with adjacent chromatograms, thereby improving separation [18,34].

The results for the simulation of the closed-loop recycling dual-mode counter-current chromatography separations with specified sample loading durations presented in Figure 4 and Figure 5 demonstrate that proper selection of operating parameters *b*, *n* and *t_s_*, *t_x_* can increase the productivity by an order of magnitude, providing the desired compound separation results. 

To promote the application of CLR DM CCC separations with specified sample loading durations, the separation simulations discussed above are presented via “Mathcad” software in the Supplementary Data.

## 5. Conclusions

In preparative and industrial-scale chromatography separations, to ensure high performance, it is necessary to load large volumes of samples and increase the concentrations in collected fractions of compounds. When separating complex mixtures containing solutes with widely different partition coefficients, this can be achieved using the CLR DM CCC method with specified sample loading durations, as described in this study. This two-stage CCC separation method is a combination of closed-loop recycling (CLR) and dual-mode (DM) counter-current chromatography. In the first stage, in CLR mode, the compounds with lower partition coefficients are separated and eluted with the mobile *x*-phase through the one end of the column. In the second stage, the compounds with higher partition coefficients are separated in the inverted-phase counter-current mode and eluted with the mobile *y*-phase through the opposite end of the column.

Although the mathematical models on which the theory of the new CLR DM CCC method is based were previously verified experimentally in [9,11,25,28,30,33], it is necessary to carry out further experimental studies for practical implementation of the CLR DM CCC method with specified sample loading durations.

## Supplementary Materials

The following are available online. The separation simulation files are available in the supplementary materials.

## References

[1] Ito Y. (2005). Golden rules and pitfalls in selecting optimum conditions for high-speed counter-current chromatography. J. Chromatogr. A.

[2] Jerz G., Winterhalter P. (2020). The 10th international conference on countercurrent chromatography held at Technische Universität Braunschweig, Braunschweig, Germany, August 1–3, 2018. J. Chromatogr. A.

[3] Friesen J.B., McAlpine J.B., Chen S.-N., Pauli G.F. (2017). The 9th International Countercurrent Chromatography Conference held at Dominican University, Chicago, USA, August 1–3, 2016. J. Chromatogr. A.

[4] Ignatova S., Sutherland I. (2015). The 8th International Conference on Counter-current Chromatography held at Brunel University, London, UK, July 23–25, 2014. J. Chromatogr. A.

[5] Morley R., Minceva M. (2020). Operating mode and parameter selection in liquid–liquid chromatography. J. Chromatogr. A.

[6] Kostanyan A.E., Belova V.V. (2019). Closed-loop recycling dual-mode counter-current chromatography. A theoretical study. J. Chromatogr. A.

[7] Kostanyan A.E., Galieva Z.N. (2019). Modeling of closed-loop recycling dual-mode counter-current chromatography based on non-ideal recycling model. J. Chromatogr. A.

[8] Zhang S., Chen H., Deng X., Chen H., Guo C., Wan L., Peng A., Chen L. (2021). Advantages of rectangular horizontal tubing in the semi-preparative counter-current chromatography bobbin. J. Chromatogr. A.

[9] Kostanyan A.E., Erastov A.A. (2016). Theoretical study of closed-loop recycling liquid-liquid chromatography and experimental verification of the theory. J. Chromatogr. A.

[10] Friesen J.B., McAlpine J.B., Chen S.-N., Pauli G.F. (2015). Countercurrent separation of natural products: An update. J. Nat. Prod..

[11] Kostanyan A., Martynova M., Erastov A., Belova V. (2018). Simultaneous concentration and separation of target compounds from multicomponent mixtures by closed-loop recycling countercurrent chromatography. J. Chromatogr. A.

[12] Mokhodoeva O., Rudik I., Shkinev V., Maryutina T. (2021). Countercurrent chromatography approach to palladium and platinum separation using aqueous biphasic system. J. Chromatogr. A.

[13] Goll J., Minceva M. (2017). Continuous fractionation of multicomponent mixtures with sequential centrifugal partition chromatography. AIChE J..

[14] Peng A., Hewitson P., Sutherland I., Chen L., Ignatova S. (2018). How changes in column geometry and packing ratio can increase sample load and throughput by a factor of fifty in counter-current chromatography. J. Chromatogr. A.

[15] Wang Y., Zhang L., Zhou H., Guo X., Wu S. (2017). K-targeted strategy for isolation of phenolic alkaloids of Nelumbo nucifera Gaertn by counter-current chromatography using lysine as a pH regulator. J. Chromatogr. A.

[16] Wang C., Sun W., Wang X., Jin Y., Zhao S., Luo M., Tong S. (2019). Large-scale separation of baicalin and wogonoside from Scutellaria baicalensis Georgi by the combination of pH-zone-refining and conventional counter-current chromatography. J. Chromatogr. A.

[17] Roehrer S., Minceva M. (2019). Evaluation of interapparatus separation method transferability in countercurrent chromatography and centrifugal partition chromatography. Separations.

[18] Kostanyan A.E., Belova V.V. (2020). Theoretical study of industrial scale closed-loop recycling counter-current chromatography separations. J. Chromatogr. A.

[19] Goll J., Morley R., Minceva M. (2017). Trapping multiple dual mode centrifugal partition chromatography for the separation of intermediately-eluting compo- nents: Operating parameter selection. J. Chromatogr. A.

[20] Ignatova S., Hewitson P., Mathews B., Sutherland I. (2011). Evaluation of dual flow counter-current chromatography and intermittent counter-current extraction. J. Chromatogr. A.

[21] Kotland A., Chollet S., Diard C., Autret J.-M., Meucci J., Renault J.H., Marchal L. (2016). Industrial case study on alkaloids purification by pH-zone refining centrifugal partition chromatography. J. Chromatogr. A.

[22] Costa F.D.N., Vieira M.N., Garrard I., Hewitson P., Jerz G., Leitão G.G., Ignatova S. (2016). Schinus terebinthifolius countercurrent chromatography (Part II): Intra-apparatus scale-up and inter-apparatus method transfer. J. Chromatogr. A.

[23] Delannay E., Toribio A., Boudesocque L., Nuzillard J.-M., Zeches-Hanrot M., Dardennes E., Le Dour G., Sapi J., Renault J.-H. (2006). Multiple dual-mode centrifugal partition chromatography, a semi-continuous development mode for routine laboratory-scale purifications. J. Chromatogr. A.

[24] Kostanyan A.E., Voshkin A.A. (2009). Support-free pulsed liquid-liquid chromatography. J. Chromatogr. A.

[25] Kostanyan A.E., Voshkin A.A., Kodin N.V. (2011). Controlled-cycle pulsed liquid–liquid chromatography. A modified version of Craig’s counter-current distribution. J. Chromatogr. A.

[26] Jeon J.S., Park C.L., Syed A.S., Kim Y.M., Cho I.J., Kim C.Y. (2016). Preparative separation of sesamin and sesamolin from defatted sesame meal via centrifugal partition chromatography with consecutive sample injection. J. Chromatogr. B.

[27] Müller M., Wasmer K., Vetter W. (2018). Multiple injection mode with or without repeated sample injections: Strategies to enhance productivity in countercurrent chromatography. J. Chromatogr. A.

[28] Kostanyan A.E., Erastov A.A. (2015). Steady state preparative multiple dual mode counter-current chromatography: Productivity and selectivity. Theory and experimental verification. J. Chromatogr. A.

[29] Kostanyan A.E. (2016). Modeling of preparative closed-loop recycling liquid–liquid chromatography with specified duration of sample loading. J. Chromatogr. A.

[30] Kostanyan A., Martynova M. (2020). Modeling of two semi-continuous methods in liquid–liquid chromatography: Comparing conventional and closed-loop recycling modes. J. Chromatogr. A.

[31] Huang X.Y., Tian M., Pei D., Liu J.F., Di D.L. (2018). Development of overlapping repeated separation of steviol glycosides with counter current chromatography and a comparison with a conventional repeated separation method. J. Sep. Sci..

[32] Kostanyan A.E., Voshkin A.A. (2007). Analysis of new counter-current chromatography operating modes. J. Chromatogr. A.

[33] Hewitson P., Sutherland I., Kostanyan A., Voshkin A., Ignatova S. (2013). Intermittent counter-current extraction-Equilibrium cell model, scaling and an improved bobbin design. J. Chromatogr. A.

[34] Kostanyan A., Voshkin A., Belova V. (2020). Analytical, Preparative, and Industrial-Scale Separation of Substances by Methods of Countercurrent Liquid-Liquid Chromatography. Molecules.

